# Low UV-C stress modulates *Chlamydomonas reinhardtii* biomass composition and oxidative stress response through proteomic and metabolomic changes involving novel signalers and effectors

**DOI:** 10.1186/s13068-020-01750-8

**Published:** 2020-06-19

**Authors:** Francisco Colina, María Carbó, Mónica Meijón, María Jesús Cañal, Luis Valledor

**Affiliations:** Plant Physiology, Department of Organisms and Systems Biology and University Institute of Biotechnology of Asturias (IUBA), University of Oviedo, Oviedo, Spain

**Keywords:** Chlamydomonas, UV-C, Biomass, ROS, Sugars, Metabolomics, Proteomics, Systems biology

## Abstract

**Background:**

The exposure of microalgae and plants to low UV-C radiation dosages can improve their biomass composition and stress tolerance. Despite UV-C sharing these effects with UV-A/B but at much lower dosages, UV-C sensing and signal mechanisms are still mostly unknown. Thus, we have described and integrated the proteometabolomic and physiological changes occurring in *Chlamydomonas reinhardtii*—a simple Plantae model—into the first 24 h after a short and low-intensity UV-C irradiation in order to reconstruct the microalgae response system to this stress.

**Results:**

The microalgae response was characterized by increased redox homeostasis, ROS scavenging and protein damage repair/avoidance elements. These processes were upregulated along with others related to the modulation of photosynthetic electron flux, carbon fixation and C/N metabolism. These changes, attributed to either direct UV-C-, ROS- or redox unbalances-associated damage, trigger a response process involving novel signaling intermediaries and effectors such as the translation modulator FAP204, a PP2A-like protein and a novel DYRK kinase. These elements were found linked to the modulation of Chlamydomonas biomass composition (starch accumulation) and proliferation, within an UV-C response probably modulated by different epigenetic factors.

**Conclusion:**

Chosen multiomics integration approach was able to describe many fast changes, including biomass composition and ROS stress tolerance, as a response to a low-intensity UV-C stress. Moreover, the employed omics and systems biology approach placed many previously unidentified protein and metabolites at the center of these changes. These elements would be promising targets for the characterization of this stress response in microalgae and plants and the engineering of more productive microalgae strains.

## Background

Plants and microalgae are well adapted to surface and underwater UV-A/B irradiation, with specific sensing systems allowing the deployment of different acclimation and photomorphogenic responses. Many of these responses involve changes in their biomass composition and their resistance to stress [1, 2]. UV-C, with a minor incidence in the natural environment, produces similar biomass, stress and development effects over plants [3, 4] and microalgae [5–7], but at a much lower dosage. Despite this, UV-C sensing and signaling mechanisms remain obscure, and is unclear if they are specific or rather shared with other stressors. Therefore, the characterization of the signal elements involved in the UV-C modulation of biomass composition and stress resistance is needed and would contribute to the generation better microalgae strains and plant varieties.

UV-C perception and signaling converge with UV-A/B in the generation of reactive oxygen species (ROS), key stress signals [8] related in turn to other signalers such as Ca^2+^, salicylic acid (SA), jasmonates (JA), and abscisic acid (ABA) [3, 4] which are known to contribute in the responses to UV among other stressors. On the other hand, in land plants such as *Arabidopsis thaliana*—hereafter Arabidopsis—and microalgae species such as *Chlamydomonas reinhardtii*—hereafter Chlamydomonas—the perception and signaling of UV-A and UV-B radiations also rely on specific photoreceptors as the UV-B specific UVR8 [1, 2]. This receptor, however, is insensitive to UV-C [9] and no specific one has been still identified for this radiation. Globally, the different signaling pathways associated to UV-B and UV-C stress responses drive transcriptional modulation programs through epigenetic mechanisms [10, 11]. Furthermore, ROS producing stresses as UV also trigger redox-based posttranslational mechanisms after the modulation of FTSH proteases [12] and translation machinery [13].

The exposure to UV-C and UV-B stresses increases plant and microalgae tolerance to stress [1, 4, 14, 15]. For UV-C stress, the increased stress tolerance is associated to the enhancement of enzymatic ROS scavenging [4, 7, 16], but also to the accumulation of valuable and protective compounds including lipids—mostly polyunsaturated fatty acids [5, 6], carotenoids [6] and sterols [7]—and UV-shielding compounds—polyphenols and flavonoids [15]. These responses resemble those induced by UV-A and/or UV-B treatments [2, 17, 18], but UV-C triggers all them at lower dosages while contributing to the rapid settling of microalgae cultures [5]. Thus, the usage of this radiation would avoid costly manipulations related to microalgae biomass harvesting and/or reduce the energy costs associated to the manipulation of their biomolecule yield and biomass composition [5]. UV-C has enhancing effects on the biomass composition of microalgae species with industrial applications such as *Haematococcus pluvialis* and *Dunaliella salina*—enhancing lipids and carotenoids synthesis—[6], and crop species—by the starch accumulation—[19–21], the molecular mechanisms behind these responses remaining unknown. The closeness of *H. pluvialis* and *D. salina* to the chlorophyte model Chlamydomonas, with its multiple available genomic/proteomic resources and simpler plant-like UV-A/B signaling pathways, makes this microalga the selected model for the characterization of this response. Then, key response proteins derived from these results, could be translated into more productive microalgae strains.

The aim of this study is to render an image of the changes in the Chlamydomonas stress response system during the first 24 h after a short and low-intensity UV-C irradiation. These changes would be identified through the description of the stress effect over microalgae physiological parameters and the integration of its proteome and metabolome changes under the initial stage of the stress response. This integration, compared to previous transcriptome and physiology-based approaches over Chlamydomonas UV-B response [1], involves novel omic data processing strategies combining the prediction of interactions between the described omic levels with existing interaction knowledge. This approach has allowed the identification of key factors related to UV-C signaling mechanisms and metabolic responses including those related to the modulation of the microalgae biomass composition.

## Results

### Physiological characterization of Chlamydomonas response to UV-C stress

**Fig. 1:**
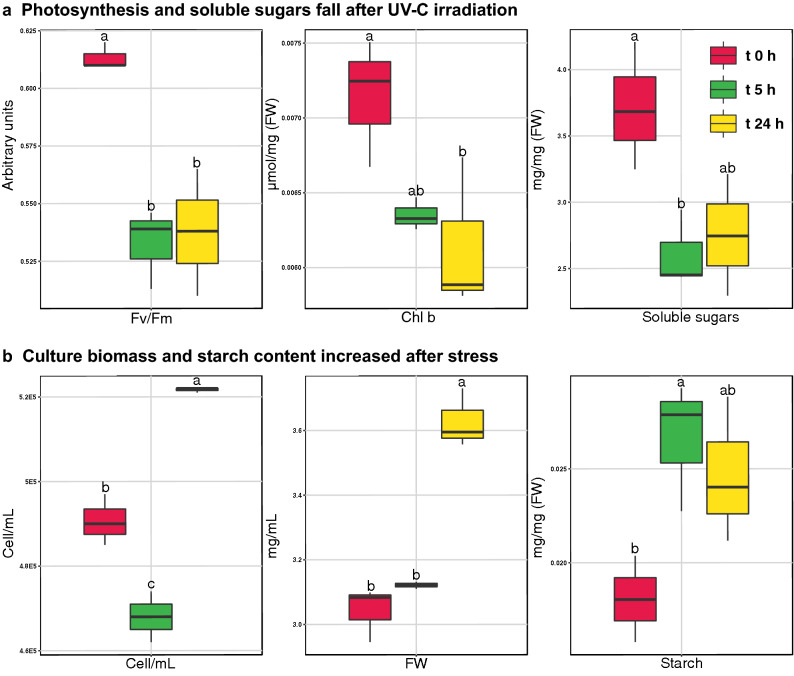
Box plot graphs showing the physiological changes on Chlamydomonas photosynthesis and biomass after UV-C irradiation. UV-C induced a decrease on the concentration of soluble sugars and photosynthetic parameters as Fv/Fm ratio and Chl b abundance (**a**). Culture biomass kept increasing after irradiation as showed the increases in cellular density (Cell/mL), culture fresh weight mL^−1^ (FW) and starch content (**b**). Only variables with significative changes (ANOVA p < 0.05) were plotted. Different letters indicate significant differences (Tukey HSD; p < 0.05)

Different physiological traits covering photosynthesis, biomass yield/composition and oxidative stress were measured along the experiment (Additional file 1: Table S1). The radiation had a negative effect over the microalgae photosynthetic parameters, with the reduction of Fv/Fm ratio and chlorophyll b concentration (ANOVA ⍺ 0.05) (Fig. 1a). Cultures cellular density and biomass (FW) kept increasing after the exposure to a low UV-C dose, despite cellular density had a transient drop 5 h after irradiation (Fig. 1b). Chlamydomonas cultures irradiated with low UV-C dosages show no reduction in their biomass yield (FW) compared to unstressed [22]. Although applied UV-C stress did not change yields, it modified Chlamydomonas biomass composition with the accumulation of starch and the fall of soluble sugars upon stress imposition (ANOVA ⍺ 0.05) (Fig. 1). This starch accumulation is also observed in UV-C-irradiated crop species such as sugar beet [19], potato [20], and lily bulb [21].

### Integrated proteomic and metabolomic responses on Chlamydomonas exposed to UV-C stress

Proteomic analysis allowed the identification of 1441 protein species in whole cell protein extracts. After data pre-processing, 885 proteins were above the abundance threshold for confident quantitation (Additional file 1: Table S2). GC–MS allowed the unequivocal identification of 69 primary metabolites, out of these 68 were considered for quantitative purposes (Additional file 1: Table S3). Detected proteins and metabolites were classified according to MapMan V4 categories [23], and 703 proteins and 54 metabolites were assigned to functional bins. These bins comprised 27 pathways for proteins and 8 for metabolites, covering different cellular processes. Out of these, 398 proteins and 14 metabolites could be considered quantitatively differential in at least one sampling time ANOVA, ⍺ 0.05 (5% FDR for protein variables) (Additional file 1: Table S2, S3).

**Fig. 2:**
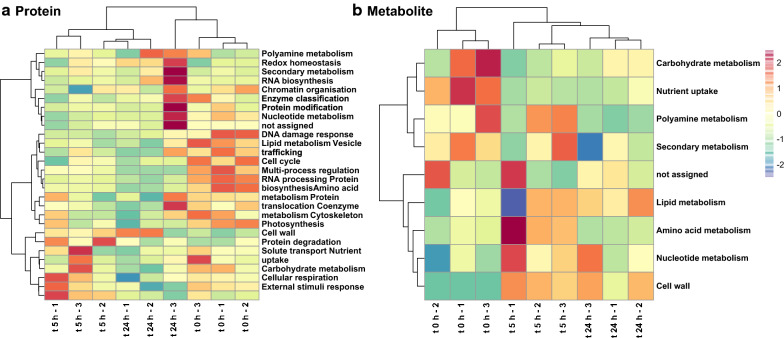
Heatmap biclustering plots over MapMan categories protein (**a**) or metabolite (**b**) total abundance. Tree was built based on Manhattan distances and Ward aggregation method. Samples clustered according to their harvesting time and differences in abundance were observed between harvesting times for some functional categories into both plots

**Fig. 3:**
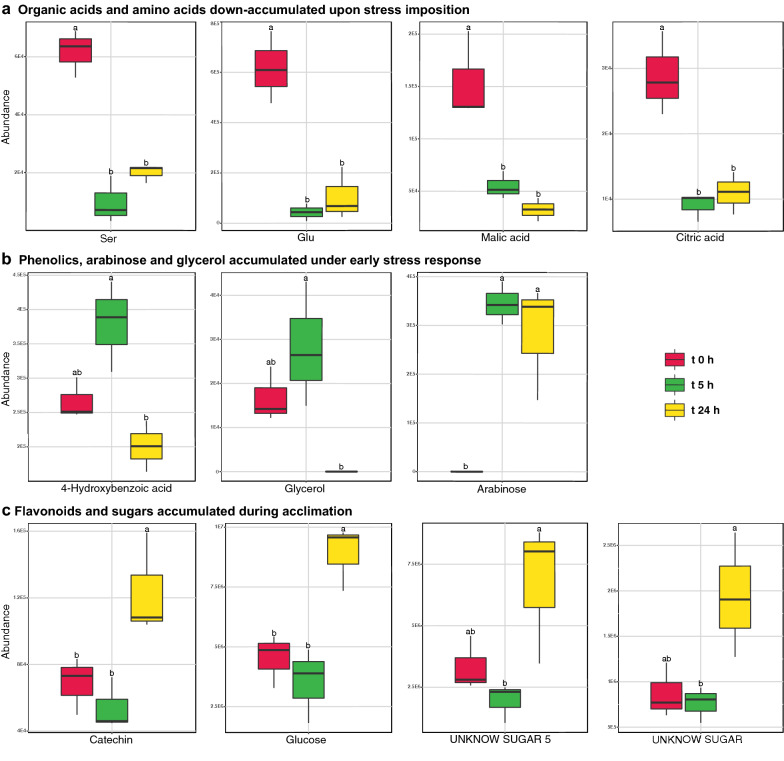
Box plot graphs showing the reshaping of the Chlamydomonas metabolome after UV-C irradiation. Metabolites related to central carbon and nitrogen metabolism (malic acid, citric acid, Ser, Glu) fall from previous high levels 5 h after UV-C irradiation (**a**). Also 5 h after stress imposition phenolics (4-hydroxybenzoic acid), glycerol and arabinose accumulated (**b**), while different sugars and flavonoids (catechin) accumulated at t 24 h (**c**). Only variables with significative changes (ANOVA p < 0.05) were plotted. Different letters indicate significant differences (Tukey HSD; p < 0.05)

Heatmap clustering based on MapMan categories distinguished the different treatments with an adequate grouping of samples (Fig. 2). At protein level (Fig. 2a) UV-C stress induced a quick reduction on multi process regulation, RNA processing, protein biosynthesis and cell cycle-related categories, all classified in the same meta-group. On the other hand, the abundance of protein degradation, external stimuli response, carbohydrate metabolism and cellular respiration categories, within the same cluster, increased after 5 h. Interestingly, redox homeostasis increased 24 h after stress start when photosynthesis bin reached its maximum abundance. Metabolites (Fig. 2b) showed a divergent distribution to those of proteins, highlighting amino acid and carbohydrate metabolism categories time shifts with their protein counterparts. Within individual metabolites, amino acids and organic acids such as serine, glutamine, and malic and citric acids down-accumulated upon stress imposition (Fig. 3a), while redox-related glycerol, arabinose and 4-hydroxybenzoic acid accumulated after 5 h (Fig. 3b). On the other hand, the increase of several unknown sugars after 24 h characterized acclimation (Fig. 3c, Additional file 1: Table S2, S3).

Samples were correctly classified by PCA when considering each omic level independently (Additional file 2: Fig. S1; Additional file 1: Table S4, S5). In proteomics dataset (Table 1, Additional file 2: Fig. S1a; Additional file 1: Table S4) principal component 1 (PC1) potentially gathered variability related to the photoacclimation to UV-C, while principal component 2 (PC2) grouped early response elements. At metabolome level (Additional file 2: Fig. S1b; Additional file 1: Table S5) PC1 explained early response with the enhancement of secondary metabolism; while PC2 joins late-accumulated sugars.

**Table 1:** List of all the high-ranking Chlamydomonas proteins in the PCA analysis with a key role in the microalgae response to UV-C stress

Accession	Loadings		q-value	Abundance	Symbol	Defline
	PC1	PC2				
Cre03.g199983.t1.1	− 0.049	− 0.010	0.000		PP2A	Ser/Thr protein phosphatase
Cre12.g558100.t1.2	− 0.050	− 0.012	0.000		PRMT2	Protein-/Histone-arginine *N*-methyltransferase
Cre07.g356600.t1.2	− 0.051	− 0.011	0.000		MINA53	MINA53 MYC INDUCED NUCLEAR ANTIGEN
Cre11.g467689.t1.1	− 0.037	0.008	0.031		PETC	Chloroplast cytochrome b6f Rieske subunit
Cre09.g393200.t1.2	− 0.006	0.066	0.000		HSP70C	Heat shock protein 70C
Cre17.g721500.t1.2	0.005	0.060	0.000		STA2	Granule-bound starch synthase I
Cre01.g022500.t1.2	0.017	0.065	0.031		MME5	NADP malic enzyme
Cre09.g394850.t1.2	0.010	0.061	0.048		TEF24	LrgB-like protein
Cre01.g045550.t1.2	0.014	0.052	0.041		APE2	Solute carrier family 35 protein
Cre13.g567600.t1.2	0.016	0.048	0.000		COX4	Mitochondrial cytochrome c oxidase subunit
Cre16.g671000.t1.2	0.018	0.065	0.000		NDA5	Type-II NADH dehydrogenase
Cre06.g261500.t1.2	0.010	0.047	0.000		CPLD58	Conserved in the Plant Lineage and Diatoms
Cre06.g274650.t1.1	0.020	0.058	0.000		NUOAF4	Complex I associated CIA30 protein
Cre01.g025250.t1.1	0.017	0.057	0.000		RFK2	Riboflavin kinase
P09144	0.023	0.062	0.003		psaB	PSI P700 chlorophyll a apoprotein A2
P06007	0.036	0.031	0.039		psbD	Photosystem II D2 protein
Cre03.g189800.t1.2	0.043	0.009	0.031		CYN38	Peptidyl-prolyl cis–trans isomerase
Cre17.g720250.t1.2	0.046	− 0.016	0.024		LHCB4	Light-harvesting protein of PSII
Cre08.g370450.t1.2	0.042	− 0.029	0.000		MGE1	Mitochondrial GrpE homolog
Cre12.g560950.t1.2	0.046	− 0.036	0.006		PSAG	Photosystem I reaction center subunit V
Cre06.g256250.t1.2	0.043	− 0.021	0.043		TEF14	Thylakoid lumenal protein
Cre03.g182551.t1.2	0.043	− 0.024	0.024		PCY1	plastocyanin, chloroplast precursor
Cre11.g476750.t1.2	0.030	− 0.060	0.048		FNR1	Ferredoxin-NADP reductase, chloroplast
Q32065	0.032	− 0.059	0.045		FTSH	Uncharacterized 341.7-kDa protein
Cre02.g084500.t1.1	0.026	− 0.056	0.000		CGL134	Possible phytol kinase
au5.g1142_t1	0.029	− 0.060	0.000		DYRK	Ser/thr protein kinase, DYRK like

Accession for each protein is followed by the protein loadings in the first (PC1) and second (PC2) components of the PCA analysis, q-value, protein abundance change between the three measured time points, and protein symbols and defline

**Fig. 4:**
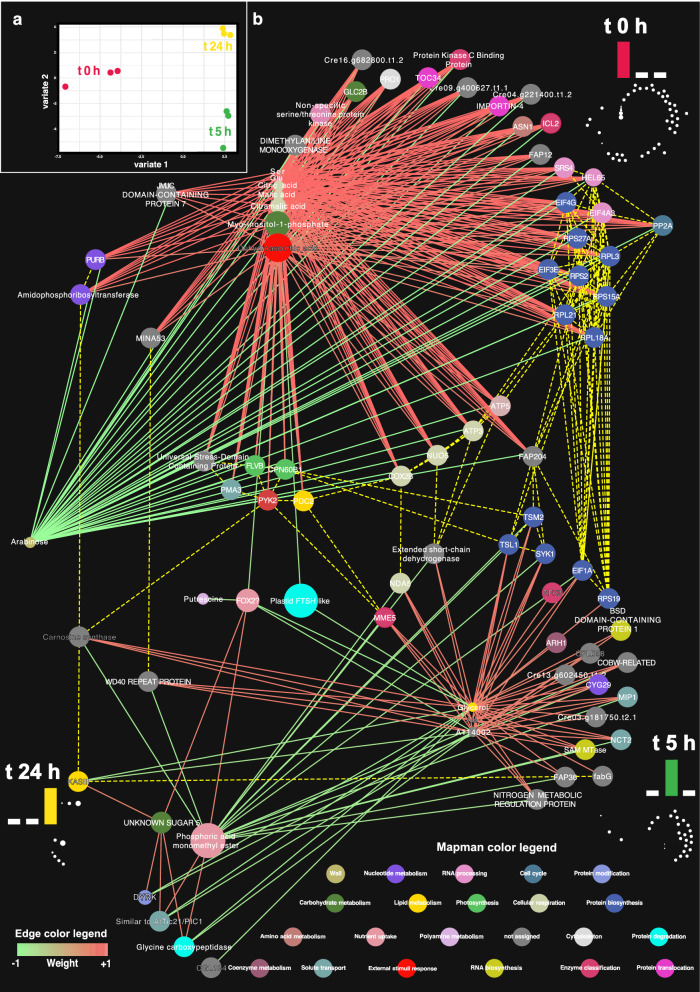
sPLS-STRING sample plot. sPLS differentiated between harvesting times (**a**). sPLS-based network overlapped with the STRING network including the protein nodes included in the sPLS-based network. Resulting network showed the response differentiation between Chlamydomonas early and acclimation responses (**b**). Network nodes were colored according to their MapMan categories linking node size and the sPLS-derived edge color to size and weight, respectively. Only the sPLS-derived edges with correlations higher or lower than ± 0.6 were visualized. Nodes without any edge above the threshold were removed from this analysis. Protein–protein STRING-derived edges were represented as dashed yellow lines

A protein–metabolite correlation network was also defined (Fig. 4b) through the integration of both datasets employing sPLS regression (Fig. 4a, Additional file 1: Table S6). This correlation network overlapped with the STRING network of the correlation network nodes. Within the resulting network sPLS-STRING (Fig. 4b) the cluster centered on early depleting metabolites (citrate, malate, glutamate, dehydroascorbate) and early accumulated arabinose gathered multiple proteins depleting upon stress start and related to translation (ribosomal subunits); cellular respiration and carbon metabolism-related PYRUVATE KINASE (PYK2), ISOCITRATE LYASE (ICL2) and multiple mitochondrial respiratory chain subunits; and development/stress-related MYC INDUCED NUCLEAR ANTIGEN (MINA53) and PHOSPHATASE 2A (PP2A) homolog Cre03.g199983.t1.1. STRING, respectively, highlighted the interaction between the depleting mitochondrial and ribosomal subunits and the links of the later to the different and also early depleting translation initiation factors. Moreover, these translation-related proteins were predicted to be associated to the PP2A homolog.

STRING linked the early depleting PP2A and ribosome subunits cluster and also translation-related FLAGELLAR ASSOCIATED PROTEIN 204 (FAP204) to different early accumulated aminoacyl tRNA synthetases (TSM2, TSL1, SYK1) and the EUKARYOTIC TRANSLATION INITIATION FACTOR 1A (EIF1a). TSM2, TSL1, SYK1 and EIF1a were included along other early (t 5 h) accumulated elements into a cluster centered on early accumulated glycerol including redox and carbon fixation RIBOFLAVIN KINASE (RFK2), GLUTATHIONE S-TRANSFERASE homolog (CPLD58), AQUAPORIN (MIP1), NDA5, and MME5; translation modulation *S*-ADENOSYL-l-METHIONINE-DEPENDENT METHYLTRANSFERASE (SAM MTase) and signaling (WD40 REPEAT PROTEIN) elements. STRING highlighted the possible interactions between the WD40 REPEAT PROTEIN and the early depleted development/stress-related MINA53. Glycerol cluster correlated negatively to another cluster with elements accumulated after 24 h centered in UNKNOWN SUGAR 5. Fatty acid (FA) synthesis Β-KETOACYL-[ACYL-CARRIER-PROTEIN] SYNTHASE III (KASIII) (Cre04.g216950.t1.2) and a DYRK like kinase (Cre01.g008550.t1.1 or au5.g1142_t1) were positively correlated to the late accumulated sugar. An uncharacterized FILAMENTOUS TEMPERATURE SENSITIVE H like (FTSH) like protease (Q32065) connected the cluster centered on early depleted elements with the clusters gathering elements accumulated after 5 and 24 h of UV-C irradiation.

Since protein–metabolite interactions in the sPLS-STRING network (Fig. 4b) were only based on mathematical models, STITCH analysis was applied to improve these interactions based on biological models (Additional file 2: Fig. S2). STITCH network highlighted the connections of early accumulated glycerol to protein biosynthesis, respiration and lipid metabolism along the mentioned STRING protein–protein interactions.

### UV-C irradiation affected mitochondrial electron transport enhancing ROS production and inhibiting oxidative phosphorylation

Mitochondrial respiration and ATP synthesis downregulated under UV-C stress with the depletion of multiple subunits of respiratory and F_1_F_0_ ATP synthase complexes, excepting the early accumulated IV subunit (COX4) (Table 1, Additional file 1: Table S2). Interestingly, UV-C treatment increased the abundance of chaperones associated to respiratory complexes. NUOAF4, chaperone of the large complex I, was exclusively detected on early stress response and HSP70C, a subunit of the IV associated HSP70 complex, peaked at this stage (onefold at t 5 h) (Table 1, Additional file 1: Table S2). HSP70 complex can aggregate with MGE1 and COX4, also up-accumulated under tested UV-C stress (Table 1, Additional file 1: Table S2), allowing the incorporation of COX4 into the IV complex [24]. Moreover, the accumulation of HSP70C has been described in response to heat stress induced protein misfolding in Chlamydomonas [25]. UV-C irradiation effect over mitochondrial proteins also triggers ROS bursts [8]. ROS scavenging catechin, catechin biosynthesis related CHALCONE ISOMERASE (Cre12.g517100.t1.1) and redox-related TCA ISOCITRATE DEHYDROGENASE (IDH2) accumulated under UV-C (0.8-, 1.5-, 2.2-fold at t 24 h) (Table 1, Additional file 1: Table S2, S3). Moreover, also redox-related RIBOFLAVIN KINASE (RFK2) was exclusively detected under early stress (Table 1, Additional file 1: Table S2). RFK overexpression protects human cells from oxidative stress-enhancing GSH metabolism [26].

### Low-intensity UV-C irradiation modulated thylakoid electron transport and fixed carbon allocation under inhibited respiration

Tested radiation damaged chloroplastic proteins, especially those of antenna and PSII as shown by the reduction of Fv/Fm ratio and chlorophyll b concentration (Fig. 1a, Additional file 1: Table S1). Enhanced damage was linked to an increase in protein turnover and protection. Central photosystem II (PSII) PsbD subunit (D2) peaked under early response along PSI P700 CHLOROPHYLL A APOPROTEIN A2 (psaB) (0.9-, 2.4-fold at t 5 h) and antennal CHLOROPHYLL a-b BINDING PROTEIN CP29 (LHCB4) up-accumulated under stress (onefold at t 24 h) (Table 1, Additional file 1: Table S2). Chlamydomonas LHCB4 is a key modulator of non-photochemical quenching (NPQ) and state transitions [27]. The accumulation of D2 and LHCB4 proteins matched the accumulation of a FTSH-like chloroplastic protease (Q32065) (Table 1, Additional file 1: Table S2), working together as a photosynthetic protein protection and turnover mechanism [28–30]. These elements accumulated along others associated to the repair and assembly of photosynthetic complexes such as the THYLAKOID LUMINAL FACTOR (TEF14), CYN38 and CHLOROPLASTIC DNAJ-LIKE PROTEIN (CDJ1) (2.7-, 2.4-, 1.2-fold, respectively, at t 24 h) (Table 1, Additional file 1: Table S2). Arabidopsis mutants on TEF14 and CYN38 orthologs fail to assembly and repair PSII [29, 31]. Chlamydomonas CDJ1 organizes chloroplast HSPs under heat stress [32] and its accumulation under UV-C pointed to an enhanced protection of photosynthetic proteins and complexes. The accumulation of CDJ1 under stress matched the early accumulation of the mitochondrial HSP70C chaperone, but no chloroplastic HSPs accumulated. Thus, these changes suggested that the tested low UV-C dosage damages PSII, triggering different measures to avoid and repair damage.

Response to chloroplastic UV-C damage also involved the accumulation of the PSI stabilizing PSI reaction center subunit V (psaG), FERREDOXIN NADP REDUCTASE (FNR1) and PLASTOCYANIN (PCY1) (2.7-, 1-, 1.6-fold at t 24 h) (Table 1, Additional file 1: Table S2). The accumulation of these proteins—involved in both linear and cyclic electron flow (LEF and CEF)—and the enhanced PSI/II turnover and protection are compatible with an enhancement on LEF and/or CEF on acclimation. In spite of this, CEF/LEF-related CYTOCHROME b6f RIESKE IRON-SULFUR CENTER SUBUNIT (PETC) down-accumulated on acclimation (− 9.6-fold at t 24 h) and Fv/Fm ratio did not recover control values after UV-C irradiation (Table 1, Fig. 1a, Additional file 1: Table S1, S2). On the other hand, LEF and CEF happens as independent processes in Chlamydomonas, where supercomplexes of PSI, Cytb6f and FNR are exclusively dedicated to the later [33]. PSI subunits and FERREDOXIN NADP REDUCTASE (FNR1) accumulated under stress and other subunits associated to this complex such as PETO, PGRL1, Cyt b6 and Cyt f maintained their abundance (Additional file 1: Table S2).

**Fig. 5:**
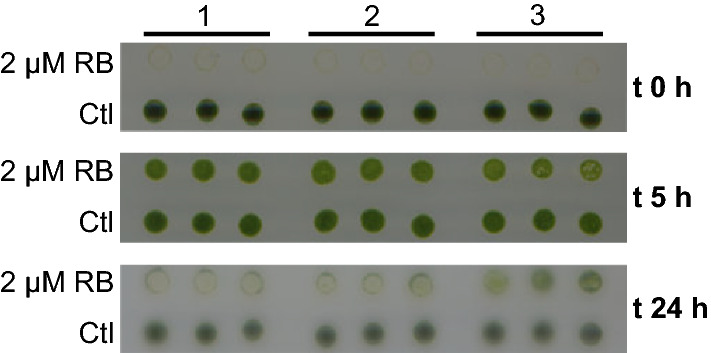
UV-C stress enhances Chlamydomonas tolerance to rose Bengal (RB)-induced singlet oxygen stress. Unstressed Chlamydomonas cultures were unable to grow after their exposure to 2 μM RB. Culture samples from the t 5 h harvest had an increased tolerance to RB-induced oxidative stress with no differences in growth between untreated and 2 μM RB-treated conditions. Tolerance to RB-induced singlet oxygen stress was reduced in culture samples from the t 24 h as the RB-treated samples grow less than the untreated ones

The fall of Fv/Fm ratio and chlorophyll b abundance, the accumulation of the NPQ/state transition-related antennal protein LHCB4, and the diverging changes in the abundance of LEF/CEF-related proteins described under tested stress matched early changes in the chloroplastic redox homeostasis. Redox modulates Chlamydomonas CEF rate, carbon metabolism and ROS scavenging mechanisms. NDA5 oxidoreductase, whose Arabidopsis homolog (NDC1) is a chloroplastic/mitochondrial NADPH-dependent quinone oxidoreductase [35, 36], and tocopherol biosynthesis PHYTOL KINASE (CGL134) were exclusively detected at t 5 and t 24 h, respectively (Table 1, Additional file 1: Table S2). Detected catalases and peroxidases, related to the detoxification of H_2_O_2_ and superoxide radicals did not change their abundance on stressed samples (Additional file 1: Table S2). Combined UV-A/B stress increases the production of superoxide radicals over singlet oxygen, which is the predominant reactive oxygen species under high light stress [37]. In spite of this, a protein containing a GLUTATHIONE-S-TRANSFERASE domain (CPLD58) was detected exclusively after 5 h of UV-C stress (Table 1, Additional file 1: Table S2). A glutathione peroxidase and a glutathione-s-transferase drive the Chlamydomonas detoxification response to the increased production of singlet oxygen under high light stress [38]. Applied UV-C dosage rapidly and transiently enhanced the tolerance of the treated cells to rose Bengal (RB)-induced singlet oxygen (^1^O_2_) oxidative stress. Cells from the t 5 h harvest were able to survive on 2 μM RB while cells from the t 24 h harvest showed an oxidative stress tolerance close to those of unstressed cells, unable to grow at that RB concentration (Fig. 5). *D. salina* and *H. pluvialis* accumulate singlet oxygen scavenging carotenoids and malondialdehyde (MDA)—a lipid peroxidation product—under UV-C stress [6]. Strawberry plants exposed to UV-C, experiment a transient increase in their antioxidant capacity [16]. Other redox regulators such as THIOREDOXIN M (TRXm) accumulated under stress (2.5-fold at t 24 h). On the other hand, redox regulated chloroplastic protein NADP MALATE DEHYDROGENASE (MME5) accumulated exclusively under early stress (fivefold at t 5 h) (Table 1, Additional file 1: Table S2). This enzyme is part of the malate shuttle, transferring not only carbon but excess reducing power from chloroplast to cytoplasm and other organelles.

The early accumulation of redox/carbon-related MME5 after UV-C irradiation concurred with the accumulation of glycerol (0.7-fold at t 5 h) (Fig. 3, Additional file 1: Table S3). Glycerol synthesis is an ATP-producing and NADH-consuming fermentative process. Moreover, glycolate/glycerate (TEF24), triose phosphate:Pi (APE2) antiporters accumulated under stress (2.9-, 4-fold, respectively, at t 5 h) and glycerol transporter (MIP1) was exclusively detected at t 5 h (Table 1, Additional file 1: Table S2). On the other side, OPP 6-Phosphogluconate Dehydrogenase (GND1), a NADPH producing enzyme, also accumulated on t 5 h (1.3-fold). Arabidopsis GND1 homolog (PGD) accumulate under ROS stress providing the required NADPH for the ROS scavenging mechanisms [39]. RuBisCO large subunit (RBCL) also accumulated 5 h after UV-C irradiation (onefold at t 5 h) (Additional file 1: Table S2). UV-B and other ROS producing stresses as O_3_ induce a decrease in the abundance of *rbc*L transcripts, its translation, and in the activity of RBCL [40]. The damage of RBCL can inhibit its translation, through the exposure of its N-terminal regions, a known inhibitor of *rbc*L translation, after the disassembly of RuBisCO holoenzyme [41]. However, low-intensity UV-B or UV-C exposure can enhance RuBisCO activity as in *D. salina* [42] and cyanobacteria [43], respectively. In Chlamydomonas, UV-C radiation depleted RuBisCO small subunit isoform 1 (RBCS1), while isoform 2 (RBCS2) abundance was not affected (Additional file 1: Table S2). In these algae both RBCS isoforms have a large equivalence [44] and, contrary to RBCL, its combined abundance is stable during cell cycle [45]. However, its particular functional differences need to be characterized, as different ratios of RBCS isoforms differentially modulate the holoenzyme carboxylation activity in plants [46]. Starch accumulated under stress (Fig. 1b) along many enzymes related to their synthesis such as GRANULE BOUND STARCH SYNTHASE (STA2) (2.1-fold at t 5 h) and STARCH BRANCHING ENZYME (SBE3), accumulated at t 5 h (Table 1, Additional file 1: Table S1, S2). UV-C enhanced starch synthesis matched the down-accumulation of soluble sugars (Fig. 1b), the late accumulation of glucose and unknown sugars such as UNKNOWN SUGAR, UNKNOWN SUGAR 4 and UNKNOWN SUGAR 5 (1-, 1.2-, 1.2-, 1-fold at t 24 h), and the depletion of TREHALOSE-6-PHOSPHATE SYNTHASE (TPS) (Table 1, Fig. 3c, Additional file 1: Table S1, S2, S3). TPS produces trehalose-6-phosphate (T6P), an inhibitor of the SnRK1 kinase, which was a key node in plant carbon metabolism also influenced by the cellular redox status [47, 48]. Sugars and starch also accumulate in UV-C treated lily bulb [21] and sugar beet [19].

Accumulation of proteases such as FTSH and photosystems subunits under tested UV-C irradiation (Table 1, Additional file 1: Table S2) suggested an increased protein turnover which could act as both a source and a sink of free amino acids. Serine and glutamate down-accumulated 5 h after irradiation (− 2.65-, − 3.83-fold, respectively) (Fig. 3a, Additional file 1: Table S3). Many enzymes related to amino acid biosynthesis such as SERINE ACETYLTRANSFERASE (SAT3), SERINE and ALANINE GLYOXYLATE TRANSAMINASES (SGA1, AGT2), ISOPROPYLMALATE DEHYDROGENASE (LEU3) accumulated (2.3-, 1.5-, 1.8-, 3.6-fold at t 5 h) and nitrogen fixation proteins such as GLUTAMINE SYNTHETASE and Fd-DEPENDENT GLUTAMATE DEHYDROGENASE remained unchanged. Other amino acid-related enzymes such as OXOPROLINASE (Cre07.g325748.t1.1) were exclusively detected on t 5 h or as CYSTATHIONINE BETA LYASE (METC) and METHYLCROTONYL CoA CARBOXYLASE alpha subunit exclusively on stressed samples (Table 1, Additional file 1: Table S2).

### Alternative signalers modulate cell proliferation, development and metabolism under UV-C

The observed changes in protein turnover/protection carbon metabolism and ROS/redox-related elements induced by UV-C were coupled to rapid changes on the abundance of many development and translation-related proteins. sPLS-STRING network (Fig. 4b) clustered many of these and linked the WD40 REPEAT PROTEIN (Cre12.g495650.t1.2)—accumulated exclusively at t 5 h, to the development related MINA53 (Cre07.g356600.t1.2)—down-accumulated under stress (Table 1, Additional file 1: Table S2). The mutation of the human homolog of MINA53, MINA53/RIOX2, inhibits DNA replication/repair mechanisms and cell proliferation in human cell lines [49]. MINA53 was also linked in the sPLS-STRING network (Fig. 4b) through C/N metabolism to JmjC protein JMJC DOMAIN CONTAINING PROTEIN 7 (Cre03.g175750.t1.2), also down-accumulated under UV-C stress. Arabidopsis homolog to JMJC DOMAIN CONTAINING PROTEIN 7 (JMJ32) is a HISTONE H3 lysine 27 (H3K27) demethylase, while its human homolog (JMJD7) is a lysyl hydroxylase. HISTONE-ARGININE N-METHYLTRANSFERASE (PRMT2) also depleted upon stress imposition (Table 1, Additional file 1: Table S2). The fluctuations in the abundance of these histone related proteins under UV-C suggested the importance of the epigenetic- and/or amino acid residue hydroxylation-based modulation mechanisms in this stress response. sPLS-STRING and STICH networks also highlighted the link of C/N metabolism and protein synthesis to early accumulated translation modulation-related elements such as EIF1a, FAP204 and aminoacyl tRNA synthases, and to a signaling PP2A like protein (Cre03.g199983.t1.1), which were exclusively detected in control samples (Fig. 4b, Table 1, Additional file 2: Fig. S2, Additional file 1: Table S2). This PP2A like phosphatase is homologous to several Arabidopsis PP2A which are key nodes in plant immunity integrating pathogen perception at membrane level with pathogen response at multiple levels including SA, ABA and TOR signaling, and C/N metabolism modulation [50, 51]. PP2As link to TOR allowing these proteins to regulate cell growth in response to the environment [52]. Thus, the connection of PP2A to protein synthesis and C/N metabolism in the sPLS-STRING and STITCH networks (Fig. 4b, Additional file 2: Fig. S2) is suggestive of the PP2A-like mediated tuning of TOR pathway in UV-C stressed Chlamydomonas. More directly related to the modulation of carbon metabolism under stress, sPLS-STRING network (Fig. 4b) highlighted a correlation between DYRK kinase (Cre01.g008550.t1.1 or au5.g1142_t1) and the accumulation of sugars and glycerol. This DYRK kinase, registered as CMGC_DYRK-PRP4 in the iTAK database [53], accumulated 24 h after UV-C irradiation start (Table 1, Additional file 1: Table S2). Other Chlamydomonas DYRK such as TAR1 and STD1 are known for their roles in carbon storage under nutrient stress [54, 55].

Results showed an early (t 5 h) Chlamydomonas response to UV-C focused on damage avoidance through enhanced singlet oxygen scavenging (Fig. 5) and protein protection/turnover (Table 1, Additional file 1: Table S2). In plants UV-C enhances ROS production and triggers ROS scavenging mechanisms [4, 8]. This early response also involved rapid changes in the cell carbon allocation with the accumulation of starch and glycerol (Fig. 1, 3). Starch accumulation remained increased 24 h after UV-C irradiation (Fig. 1) and carbon metabolism was further modulated as specific sugars were also accumulated (Fig. 3). Late accumulated sugars and starch matched the up-accumulation of a DYRK kinase (Cre01.g008550.t1.1) (Table 1) whose homologs regulate carbon fluxes in Chlamydomonas [54, 55]. Moreover, these responses were linked to protein expression/epigenetic modulation elements such as MINA53 and PRMT2 that might be driving the proteogenomic changes after the observed UV-C adaptation and enhanced oxidative stress resistance in Chlamydomonas. All these elements would help in further UV-C stress characterization or exploitation towards the generation of enhanced strains.

## Discussion

### Chlamydomonas response to UV-C stress relies on redox modulation

**Fig. 6:**
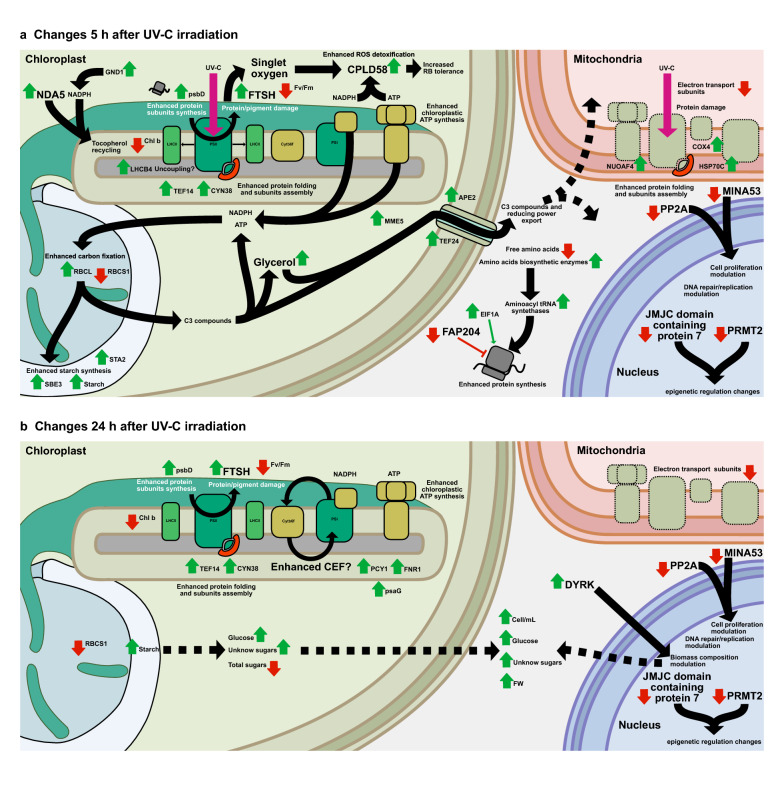
Summary of the major changes in the metabolism and physiology of *C. reinhardtii* 5 and 24 h after UV-C irradiation. At 5 h glycerol accumulated along the probable enhancement of chloroplast NADPH/ATP synthesis, carbon fixation (RBCL), fixed carbon storage and transport (STA2, SBE3, MME5, TEF24, APE2), singlet oxygen detoxification (CPLD58) and chloroplastic protein damage (FTSH). These changes were associated to a cell-wide increase in protein synthesis (FAP204). At 24 h there was a probable increase of CEF, and the culture FW increased along its content in different sugars. A novel DYRK kinase was probably associated to these biomass changes, along other stress signaling elements (JMJC domain containing protein 7, PRMT2, MINA53, PP2A). Legend: continuous or dashed black lines, respectively, indicate known or potential interactions between linked biomolecules. Green and red arrows, respectively, indicate up or down accumulation during the stress. UV-C specific are differentiated from other UV-induced responses by using a larger font size

Plants and also algae are exposed to ever changing light environments and are continuously forced to adapt. In this work, we have described Chlamydomonas acclimation to UV-C stress, a process focused on damage avoidance and repair mechanisms centered on photosynthesis and redox/energy metabolism modulation, and on the enhancement of protein turnover and ROS scavenging. Tested UV-C radiation damaged Chlamydomonas photosystems as shown by the early downregulation of Fv/Fm ratio, the reduction of chlorophyll b abundance and the evidence of an enhanced PSI/II protein turnover, protection and assembly. The accumulation of LHCB4, a central PSI/II protein related to UV-sensing and state transition, supports the radiation effect on photosynthesis and the microalgae response focused on photoprotection and protein turnover. ROS detoxification response is enhanced under UV-C complementing protein turnover with the aim of preventing protein misfunction [7]. UV-A/B stress has been described as a generator of superoxide over other oxygen species as singlet oxygen, common in high light stress [37]. Interestingly, tested low UV-C dosage enhanced Chlamydomonas tolerance to exogenous RB induced singlet oxygen stress as well as the accumulation of possible singlet oxygen enzymatic scavengers as CPLD58, over H_2_O_2_ and superoxide scavenging enzymes. These changes suggested the importance of singlet oxygen stress at low UV-C dosages (Figs. 5, 6, Additional file 1: Table S2). The generation of singlet oxygen is tightly related with the radiation effects over the chloroplast redox status and the chlorophylls state [38]. On the other hand, the scavenging of these oxygen species also relies on the chloroplast ability to generate ATP and reduced equivalents. The enhancement of chloroplast ATP synthesis would be necessary as UV-C downregulated the mitochondrial electron transport and oxidative phosphorylation. The early enhancement of chloroplastic ATP and NADPH-producing pathways under UV-C, such as glycerol synthesis and NADPH-producing OPP enzyme GND1, and the possible late increase of CEF support the modulation of the chloroplastic energy/redox metabolism under this stress. These changes were tightly connected to the modulation of carbon fixation, metabolism and transport (Figs. 1, 2, 3, 4 and 5, Additional file 1: Table S1, S2, S3). In Arabidopsis, the accumulation of ROS quenchers such as tocopherol, was revealed as a scavenging mechanism contributing to the maintenance of PSII functionality under high light [56]. The differential accumulation of enzymes related to the synthesis of tocopherol, the scavenging of ROS species (singlet oxygen), and the modulation of reduced equivalents (NADPH) and thylakoidal reduced plastoquinone (PQH_2_) pools summarize the Chlamydomonas response to the increase of ROS and the breakage of redox homeostasis under UV-C stress.

UV-C induced non-enzymatic ROS scavenging mechanisms in Chlamydomonas through the accumulation of diverse phenolic compounds (4-hydroxybenzoic acid, catechin) and phenolic metabolism enzymes such as CHALCONE ISOMERASE, but also through the late accumulation of sugars, and the enhancement of tocopherols synthesis as suggested by the accumulation of a predicted PHYTOL KINASE (CGL134) (Fig. 3, Additional file 1: Table S2, S3). Phenolic compounds and tocopherol are also accumulated by *Pinus radiata* plants irradiated with UV-A/B/C [57]. The accumulation of soluble sugars is observed under different plant abiotic stresses associated to a ROS scavenging function [58, 59]. Arabidopsis PHYTOL KINASE 1 (VTE5) participates in tocopherol synthesis through the recycling of phytol from degraded chlorophyll [60], thus the presence of a PHYTOL KINASE under UV-C also suggest UV/ROS damage to the antenna pigments. Photosynthetic antenna damage is commonly pointed out by an increase in the Chla/b ratio after the reduction of chlorophyll b abundance, exclusively present into the antenna complexes [61]. Interestingly, applied UV-C stress did not affect Chla/b ratio, but reduced chlorophyll b content (Fig. 1a, Additional file 1: Table S1) and, in consequence, chlorophyll. This reduction of chlorophylls under tested UV-C dosage could be inducing subtle reduction/degradation of the antenna complexes. Phytol tails from degraded chlorophyll might be contributing to ROS scavenging, working along other photoprotective mechanisms (Fig. 6). Applied irradiation also modulated Chlamydomonas starch content, which increased along sugars, pigments and protective compounds. The accumulation of starch is shared with land plants, as it is also accumulated by many crops irradiated with UV-C [19–21] while most microalgae accumulate lipids instead [5–7].

The scavenging/stabilizing function of phenolics, sugars and tocopherol are complemented by the activity of also early induced oxidoreductase NDA5 (Additional file 1: Table S2). The Arabidopsis homolog to this protein, NDC1, reduces oxidized plastoglobuli tocopherol and plastoquinone on NADPH. The reduced plastoquinone (PQH_2_) into plastoglobuli can easily diffuse back to thylakoidal membranes [56, 62] participating in a redox buffering process which can also contribute to ATP synthesis through the cycling of electrons around PSI. Thus, PQH_2_ would be acting as a central redox balancer which modulates both chloroplastic electron transport rate and chloroplastic ROS scavenging mechanisms.

The enhancement of NDA5 redox modulator under UV-C is complemented by an early increase in carbon fixation, a NADPH consuming process, and the also early efflux of photosynthetic carbon and reducing power from chloroplast. The increase in carbon fixation was supported by the t 5 h accumulation on RBCL and the maintenance of RuBisCO ACTIVASE (RCA1) and RuBisCO SMALL SUBUNIT 2 (RBCS2) levels suggesting an early increase in RuBisCO activity (Additional file 1: Table S2). The differential behavior of the RBCS1 subunit (Additional file 1: Table S2) suggests a possible holoenzyme configuration change under stress. However, the functional consequences of differential abundance of RBCS isoforms are still unknown [46], although these isoforms have differential interactomes [63]. The possible holoenzyme change observed under UV-C would contribute to adapt the RuBisCO activity to the changing cell environment. Some plant RBCS subunits also show a differential expression pattern contributing to the modulation of RuBisCO carboxylase activity under different CO_2_ concentrations [46]. Moreover, RBCS have a key role in organisms with carbon concentrating mechanisms (CCM) contributing to the assembly of RuBisCO complexes within the pyrenoid [64]. Thus, changes in the abundance of specific RBCS isoforms would also have consequences on the microalgae CCM. The increase in RuBisCO activity and the early accumulation of glycerol, starch and starch synthesis enzymes supports the enhancement on carbon fixation, coupled to its export to the cytoplasm as suggested by the early accumulation of NADPH-dependent malate shuttle (MME5) and triose phosphate:Pi (APE2) transporter (Fig. 6, Additional file 1: Table S2, S3). RuBisCO upregulation has been described under low UV-B in microalgae [42] and low UV-C in cyanobacteria [64]. While NDA5 and carbon fixation can avoid the overreduction of the photosynthetic electron chain at chloroplast level, carbon export would act as a shuttle moving carbon and reducing power in the form of malate and glycerol to and from other cellular compartments [65, 66], including the mitochondria, where they can contribute to generate ATP and avoid the overreduction associated to the inhibition of its electron transport chains (Additional file 1: Table S2). These early redox unbalances would be after the accumulation of subunits associated to the *Chlamydomonas* CEF supercomplexes (Additional file 1: Table S2), suggesting the late enhancement of CEF under late UV-C acclimation, an ATP-producing process (Fig. 6). Chlamydomonas has PSI, CYTB6F and FNR exclusively dedicated to CEF which assemble under reducing conditions and perform Fd-CEF electron transfer, where FNR consumes NADPH reducing ferredoxin and contributing to the production of ATP and regenerating NADP+ [33, 34]. These elements would be part of a larger PSII protection/repair mechanism associated to its uncoupling and the production of needed ATP both for this repair and the avoidance of further damage by ROS. The possible CEF enhancement on UV-C acclimation matched the accumulation of multiple sugars and the phenolic catechin and the fall on early enhanced C3 transport suggesting the recovery of the cell redox balance (Fig. 2, Additional file 1: Table S2, S3).

### Proteome reorganization is a key process to respond to UV-C stress

As proteins are extensively damaged by UV, standing under UV radiation requires the enhancement of protein turnover mechanisms involving the degradation of damaged proteins, the synthesis of new ones and their fast and coordinated folding and integration into different complexes [67]. PSII is one of the most affected protein complexes under UV with the radiation damaging its central subunits and activating specific responses based on its turnover. The early accumulation of mitochondrial stress chaperone HSP70C subunit, linked to increased mitochondrial protein damage in Chlamydomonas [25], and chloroplast HSP organizer CDJ1 support a global increase in protein damage under UV-C stress. Moreover, also the early accumulation of enzymes involved in amino acid synthesis and the down-accumulation of Ser and Glu, probably funneled towards protein synthesis, supports an enhanced protein turnover (Fig. 6, Additional file 1: Table S2, S3). The activation of the specific PSII turnover response under UV-C was supported by the accumulation of a FTSH-like protease—FTSHs are associated to damaged PSII under combined UV-A/B/C stress [57]—central PSII subunits such as psbD, and different proteins related to the correct folding, ensemble, and repair of photosystem II such as TEF14 and CYN38 (Fig. 6, Additional file 1: Table S2).

Either the synthesis of new proteins to substitute damaged, or the modulation of the proteome to acclimate to the new conditions, requires a controlled enhancement of protein synthesis which is modulated at translation, transcription, and epigenetic levels [68]. The up-accumulation of translation initiation complex promoters such as EIF1A and aminoacyl tRNA synthetases, and the down-accumulation of a possible repressor of this complex (FAP204) under stress suggest a possible balance towards an enhanced translation activity consequence of the applied UV-C irradiation (Fig. 6, Additional file 1: Table S2). FAP204 defined as a possible repressor as its human homolog, OLA1, inhibits translation initiation complex formation [69]. Interestingly, the three translation-related elements were clustered in the protein interaction networks suggesting a common regulation after UV-C exposure (Fig. 5, Additional file 2: Figure S2). The changes in translational activity are always a reflex of prior transcriptional and epigenetic changes, thus same changes could be happening under UV-C stress. Epigenetic changes were represented by the early depletion of the histone demethylase JMJC DOMAIN CONTAINING PROTEIN 7 and the histone methyltransferase PRMT2 (Fig. 6, Additional file 1: Table S2). Arabidopsis homologs to these, JMJ32 and PRMT10, are linked to environment based FLC modulation [70, 71]. Thus, their depletion in the microalgae would be related to the epigenetic control of stress response genes under UV-C. Ultimately, transcription linked epigenetic modulation with the observed changes in translational activity. RNA processing-related S-ADENOSYL-l-METHIONINE-DEPENDENT METHYLTRANSFERASE (SAM MTase) and WD40 REPEAT PROTEIN, accumulated under UV-C stress, were linked to the early accumulated and redox/energy metabolism-related glycerol in the sPLS-STRING analysis (Fig. 5; Additional file 1: Table S2, S3). Glycerol and many of the metabolites involved in its metabolism, such as glycerol-3-phosphate, have known signaling roles in yeast [72] and plants [73].

### Novel signalers are involved in the UV-C-induced modulation of Chlamydomonas metabolism

The early response of Chlamydomonas to the UV-C irradiation was mainly based on protein protection and redox modulation to limit further direct UV and ROS damage. This response was probably coupled to ROS-based signaling pathways as in land plants [4, 74], although no evidences were found of the involvement of salicylic and jasmonic acids, common plant ROS signalers upregulated in UV-C-stressed plants [3].

Regardless of the still unknown microalgae UV-C perception and signaling mechanisms the omic and systems biology approach to this stress linked many proteins and metabolites, some of them previously unidentified as CPLD58 and the novel FTSH protease, to the observed UV-C effect over Chlamydomonas photosynthesis, protein turnover, biomass and cell proliferation (Fig. 1a, b). The translation modulator FAP204 and the cell proliferation-related PP2A-like and MINA53 proteins are also examples of previously unidentified proteins which could be, respectively, associated to the enhanced protein turnover under UV-C and to the modulation of the culture growth, which was transiently reduced 5 h after stress imposition. On the other hand, the acclimation accumulated and also previously unidentified DYRK kinase was associated in the sPLS-STRING network to the late accumulated sugar (UNKNOWN SUGAR 5) pointing to its role in the observed modulation of biomass composition under UV-C (Figs. 5, 6), as other DYRK kinases modulate starch and oil accumulation in stressed Chlamydomonas [55]. This work has identified novel elements related to translation, transcription and epigenetic mechanisms which can be considered promising targets for the further characterization of the UV-C response or the exploitation of the UV metabolic modulation mechanisms towards the engineering of more productive strains.

## Conclusion

Chlamydomonas response to UV-C stress is based on the avoidance of protein damage requiring globally from the enhancement of protein protection/turnover and ROS scavenging and the modulation of photosynthesis and redox (Fig. 6). The Chlamydomonas early response to UV-C irradiation is mainly based on protein turnover/protection, singlet oxygen focused ROS scavenging and metabolic/redox modulation through the enhancement of carbon and reducing power exchange between cellular compartments. This fast response is coupled to probably ROS- and macromolecule damage-mediated signaling that would be driving a fine UV-tuned control of cell proliferation, gene expression and protein translation before Chlamydomonas late and more complex acclimation responses. Acclimation was related to specific proteome changes focused on the photosystems modulation, uncoupling PSII, enhancing CEF and the accumulation of probably ROS scavenging sugars and starch. Some of the early found and previously unknown response signalers such as translation modulator FAP204, cell proliferation-related PP2A-like protein and MINA53, and late acclimation metabolic modulator DYRK have been identified as promising targets for the further characterization of the UV-C microalgae response, including the modulation of the biomass content. Similarities between the sugar focused UV-C response of Chlamydomonas, and those previously described in crop species make this work applicable to improvement strategies aimed at a broad range of species as plants and microalgae.

## Methods

### Strains and cultures

*Chlamydomonas reinhardtii* CC-503 cw92 cultures were grown on a culture chamber (25 °C, 120 rpm, continuous 85 µE m^2^ s^−1^ irradiance provided by Sylvania GroLux lamps) in HEPES acetate phosphate (HAP) culture media [75]. HAP was formulated from Tris acetate phosphate (TAP) [76], substituting Tris for HEPES as a mass spectrometry (MS)-compatible buffer. To induce UV stress, cultures were irradiated with a UV-C dose of 0.1 W m^−2^ during the first 15 min upon experiment start. UV-C was provided by PHILIPS TUV 36W/G36 T8 lamps with a discrete UV-C emission peak at 250 nm. Initial culture was prepared 48 h before experiment start diluting 20-fold a seed culture that originated from a single colony. After 48 h, cells were harvested and diluted to 5·10^5^ cell/mL splitting the resulting culture volume between nine flasks.

### Physiological measurements

**Fig. 7:**
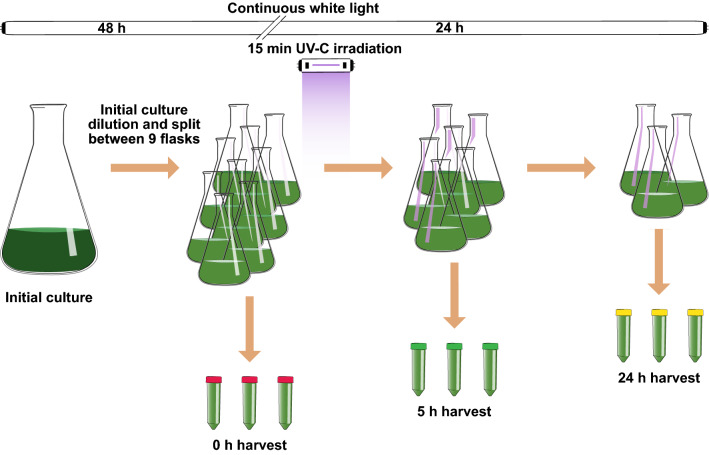
Experimental design. The initial Chlamydomonas culture grown under control conditions was diluted and split between nine flasks just before experiment start. Flasks were placed under continuous UV-C light irradiation during the first 15 min upon experiment start. Three different flasks were sampled for each harvesting time. Harvesting was performed at the experiment start (t 0 h), 5 and 24 h after UV-C irradiation start

Three flasks were sampled on each harvesting time, placed at the experiment start (0 h) and after 5 and 24 h of UV exposure (Fig. 7). Cell density was measured on each harvesting time by measuring the culture absorbance at 630 and 750 nm using the Nabi UV/vis Nano Spectrophotometer (MicroDigital Co., Korea) and the cultures photosynthetic rate (Fv/Fm) was measured with an imaging/pulse–amplitude modulation fluorimeter (OS1-FL, Opti-Sciences). Parallelly, 150 mL of culture were taken from each harvested flask and divided in three 50-mL aliquots which were centrifuged at 4000×g. Pellets fresh weight (FW) was calculated gravimetrically and then immediately frozen in liquid nitrogen. One of the three pellets harvested from each flask was employed for the combined extraction of metabolites and proteins, according to Valledor, et al. [77]. The second pellet was employed for the quantification of pigments (chlorophyll a, b and carotenoids) according to Sims and Gamon [78]. Starch, soluble sugars, free amino acids, lipid peroxidation and phenolic compounds were measured from the remaining pellet using a novel colorimetric protocol López-Hidalgo, C (unpublished) using the Nabi UV/vis Nano Spectrophotometer (MicroDigital Co., Korea). Briefly, this protocol starts from a single Chlamydomonas extract resulting from the homogenization of the cell pellet in ethanol 80%. Aliquots from this extract were employed in different ethanol-based colorimetric protocols derived from the combination and modification of previous methods for the quantification of starch and soluble sugars [79, 80], free amino acids [81], phenolic compounds [82] and the lipid peroxidation product MDA [83].

### Bengal rose drop assay

Two milliliter samples taken from each harvested flask were adjusted to 5·10^5^ cell/mL and divided in two 1-mL aliquots. 2 μM rose Bengal (RB) (Merck, Germany) was added to one of the aliquots into each pair. Resulting RB containing and control aliquots were challenged during 30 min under constant light and then three drops of 3 μL from each one were plated onto a HAP agar plate and maintained under constant light for 48 h.

### Quantitative proteome analysis (GeLC-LTQ-Orbitrap MS)

The preparation of protein samples for MS/MS analysis was performed following Valledor et al. [84] recommendations. Ten micrograms of trypsin-digested peptides were loaded per injection into a one-dimensional nano-flow LC-Orbitrap/MS and resolved in a 90-min gradient from 5 to 40% (v/v) acetonitrile/0.1% (v/v) formic acid using a monolithic C18 column (Chromolith RP-18r, Merck, Darmstadt, Germany). MS analysis was performed on an Orbitrap LTQ XL mass spectrometer [75].

Raw data coming from Orbitrap were searched with Proteome Discoverer version 2.1 (Thermo) SEQUEST algorithm as previously described [85], employing a label-free quantification based on precursor’s areas. Chlamydomonas 5.5 protein (18750 accessions), Chlamydomonas chloroplast & mitochondria (84 accessions) and Swissprot-viridiplantae (36097 accessions) databases were employed for protein identification. Only high-confidence proteins (at least one significant peptide, XCorr > 1.8, FDR 5%) present in all the biological samples of at least one treatment were considered for this analysis.

### Metabolite GC–MS analysis

Polar fraction analysis were carried out following Valledor, et al. [84] protocol with some minor changes on a triple quad instrument (TSQ Quantum GC; Thermo). In brief, 1 µL of sample was injected, and GC separated into a HP-5MS capillary column (30 m 0.25 mm) (Agilent Technologies). Oven temperature was increased from 80 °C to 200 °C at a 3 °C per min rate and then reduced to 25 °C at 10 °C per min and maintained at 25 °C for 3 min, followed by 4 min of post-run conditions at 30 °C. Mass spectrometer was operated in electron impact (EI) mode at 70 eV in a scan range of m/z 40–600. The identification of metabolites was based on the spectral characteristics and GC retention times of each individual metabolite through its comparison with the retention times and spectral characteristics of standards available in our in-house library and in Golm Metabolome Database [86].

### Biostatistical analyses

R v3.3 (R Core Team 2019) [87] software core functions and pRocessomics package (available at https://github.com/Valledor/pRocessomics) were used for all performed statistical procedures.

Proteomics and metabolomics datasets missing values were imputed through a sequential K-Nearest Neighbor algorithm. Imputation was performed only when one value per sampling point was missing. Protein and metabolite abundances were re-estimated afterwards following a sample-centric approach (individual peak areas divided by total peak area per sample). Data were then subjected to univariate (one-way ANOVA α 0.05, 5% FDR for protein variables, followed by a Tukey HSD post hoc test in all cases) and multivariate analyses: principal component analysis (PCA), heatmap clustering and sparse partial least squares regression analysis (sPLS).

sPLS-based multivariate models [88] were employed after a tuning process selecting the best combination of protein and metabolite variables to build correlation networks employing the R package mixOmics [89] using proteins as predictors and metabolites as response. Resulting correlation network was filtered keeping edges with correlation values above 0.6. Correlation network nodes were used as input to build known protein–protein and protein–protein/protein–metabolite interaction networks into the STRING and STITCH platforms, respectively [90]. Resulting networks were visualized and processed in Cytoscape V3.6.1 [91], including the overlapping of the sPLS and STRING networks. Cytoscape plug in StringApp [92] was used for STRING and STICH network import, setting interaction confidence to 0.4 (medium confidence).

## Supplementary information

**Additional file 1: Table S1.** Physiological parameters related to photosynthesis –chlorophyll a (Chl a), chlorophyll b (Chl b), carotenoids (Car), total chlorophyll content (Chl a + b), Fv/Fm and Chla/b ratios-, culture total biomass-average cell density (cell/mL) and fresh weight per mL of culture (FW)-, biomass composition-Lipid weight (LW), starch, soluble sugars, free amino acids and phenolics abundance- and oxidative stress-related malondialdehyde (MDA) content. Pigment (Chl a, Chl b, Car), biomass composition measures (LW, starch, free amino acids, soluble sugars and phenolics) and MDA abundance were expressed as µmol, mg and nmol per mg of FW respectively. For each parameter is represented the harvesting time mean and SD. Significative differences were detected through a one-way ANOVA (⍺ = 0.05) over z-centered data. Tukey HSD post hoc test (⍺ = 0.05) was performed in order define differences between the different harvesting times. **Table S2**: List of the 885 quantified proteins with their abundances estimated by the integration of precursor’s areas. Protein are designated with their respective Chlamydomonas JGI v5.5, Viridiplantae-UniProt or Augustus database accession. Percent of coverage, number of unique peptides used for identification, residues and protein molecular weight are indicated. Displayed data underwent filtering, imputation and sample abundance-based balancing. The mean abundance ± SD for each sampling time is indicated as well as their ANOVA p- and q-values (5% FDR), and the post hoc Tukey HSD test p-values calculated over Log10 transformed data. Deflines, symbols and MapMan bins were manually curated. **Table S3:** List of the 68 quantified metabolites. Metabolite names were included along their Golm metabolome database identifiers used as the main names of uncharacterized compounds. Retention time (RT) was included along the mass/charge ratios (m/z 1 and m/z 2) of the two most characteristic fragmentation ions for each compound. Metabolite abundance was estimated from the peak areas of the indicated characteristic ions. Abundance data underwent filtering, imputation and sample abundance-based balancing. The mean abundance respect to control ± SD for each sampling time are indicated as well as ANOVA p-values and post hoc Tukey (HSD) test p-values calculated over z-centered data. MapMan bins and deflines were manually curated. **Table S4:** PCA of the proteome dataset. Scores for the nine generated components for each sample are showed along proportion of the total sample variance explained by each component and loadings relating each protein variable contribution to each generated component. Proteins were identified by their respective Phytozome v5.5, Viridiplantae-UniProt or Augustus identifier. **Table S5:** PCA of the metabolome dataset. Scores of the nine generated components for each sample are showed along proportion of the total sample variance explained by each component and loadings relating each metabolite variable contribution to each generated component. Metabolites were identified by their respective names or their Golm metabolome database identifiers. **Table S6:** sPLS-based Integration proteome and metabolome datasets. Model was tuned keeping 125 protein (X) and 9 metabolite/physiology (Y) variables. Loadings relating each keep protein (X) or metabolite and physiological variables (Y) contribution to each two generated components are listed along the variable identifiers Proteins were identified by their respective Phytozome v5.5, UNIPROT-Viridiplantae or Augustus identifier. Metabolites were identified by their respective name or their Golm database identifier.

**Additional file 2: Fig. S1.** PCA biplots over z-score transformed data from protein (A) and metabolite (B) showing divergence between samples from different harvesting times and the correlation of each individual variables to each displayed component. Protein PCA (A) principal component 1 (PC1) potentially gathers variability related to the photoacclimation to UV-B/C with photosynthesis PHOTOSYSTEM II D2 PROTEIN (psbD) and CHLOROPHYLL A-B BINDING PROTEIN (LHCB4) as some of its highest rank elements. Principal component 2 (PC2) collects early response elements with signaling. WD40 REPEAT PROTEIN (Cre12.g495650.t1.2), carbon metabolism a redox-related NADP MALIC ENZYME 5 (MME5) and TYPE II NADH DEHYDROGENASE (NDA5) and protein damage-associated HEAT SHOCK PROTEIN 70C, E (HSP70C, HSP70E) had the highest loadings within this component. At metabolome level (B), PC1 explain early response with phenolic metabolism protocatechuic and 4-hydroxybenzoic acids; while PC2 joins late accumulated sugars and UV shielding catechin, both related to acclimation. Bigger dots represent individual samples and were colored according to their harvesting time (0, 5, 24 h), while small dots represent individual variables and were colored according to their MapMan category. **Fig. S2.** sPLS based STITCH network. STITCH network nodes were colored according to their MapMan categories and shaped according to the omic level element they represented (circle for proteins, quadrangle for metabolites). Edge color was link to interaction confidence (STITCH interaction score). Confidence threshold was set at 0.4 (medium confidence). Network highlighted C3 derived glycerol redox valve central role under UV-C, between protein biosynthesis, respiration modulation, lipid metabolism and oxidative stress response. Same network connected translation related EIF1a, PP2A like (Cre03.g199983.t1.1) and FAP204 to C/N metabolism and translation modulation. Translation/development related MINA53 was also connected in this network to unknown WD40 repeat protein.

## Data Availability

The datasets used and/or analyzed during the current study are available from the corresponding author on reasonable request. The Chlamydomonas JGI v5.5, Viridiplantae-UniProt or Augustus database accessions for all proteins referred in the manuscript are available in the Additional file 1: Table S1 and Table 1. Golm database identifiers for all metabolites referred in the manuscript are available at Additional file 1: Table S2.

## Abbreviations

ABA: Abscisic acid
CEF: Cyclic electron flow
HAP: HEPES acetate phosphate
JA: Jasmonates
LEF: Linear electron flow
PSI: Photosystem I
PSII: Photosystem II
PC1: Principal component 1
PC2: Principal component 2
PCA: Principal component analysis
ROS: Reactive oxygen species
PQH2: Reduced plastoquinone
SA: Salicylic acid
sPLS: Sparse partial least squares regression analysis
TAG: Triacylglycerols
TAP: Tris acetate phosphate
OEC: Oxygen evolving complex
