# Supplementary material for: Low UV-C stress modulates Chlamydomonas reinhardtii biomass composition and oxidative stress response through proteomic and metabolomic changes involving novel signalers and effectors
Source: Biotechnol Biofuels. 2020 Jun 19;13:110. doi: 10.1186/s13068-020-01750-8 (PMC7305600; doi:10.1186/s13068-020-01750-8)
Supplement: Supplementary file 2 — Additional file 2: Fig. S1. PCA biplots over z-score transformed data from protein (A) and metabolite (B) showing divergence between samples from different harvesting times and the correlation of each individual variables to each displayed component. Protein PCA (A) principal component 1 (PC1) potentially gathers variability related to the photoacclimation to UV-B/C with photosynthesis PHOTOSYSTEM II D2 PROTEIN (psbD) and CHLOROPHYLL A-B BINDING PROTEIN (LHCB4) as some of its highest rank elements. Principal component 2 (PC2) collects early response elements with signaling. WD40 REPEAT PROTEIN (Cre12.g495650.t1.2), carbon metabolism a redox-related NADP MALIC ENZYME 5 (MME5) and TYPE II NADH DEHYDROGENASE (NDA5) and protein damage-associated HEAT SHOCK PROTEIN 70C, E (HSP70C, HSP70E) had the highest loadings within this component. At metabolome level (B), PC1 explain early response with phenolic metabolism protocatechuic and 4-hydroxybenzoic acids; while PC2 joins late accumulated sugars and UV shielding catechin, both related to acclimation. Bigger dots represent individual samples and were colored according to their harvesting time (0, 5, 24 h), while small dots represent individual variables and were colored according to their MapMan category. Fig. S2. sPLS based STITCH network. STITCH network nodes were colored according to their MapMan categories and shaped according to the omic level element they represented (circle for proteins, quadrangle for metabolites). Edge color was link to interaction confidence (STITCH interaction score). Confidence threshold was set at 0.4 (medium confidence). Network highlighted C3 derived glycerol redox valve central role under UV-C, between protein biosynthesis, respiration modulation, lipid metabolism and oxidative stress response. Same network connected translation related EIF1a, PP2A like (Cre03.g199983.t1.1) and FAP204 to C/N metabolism and translation modulation. Translation/development related MINA53 was also connected in th [file 13068_2020_1750_MOESM2_ESM.pdf]

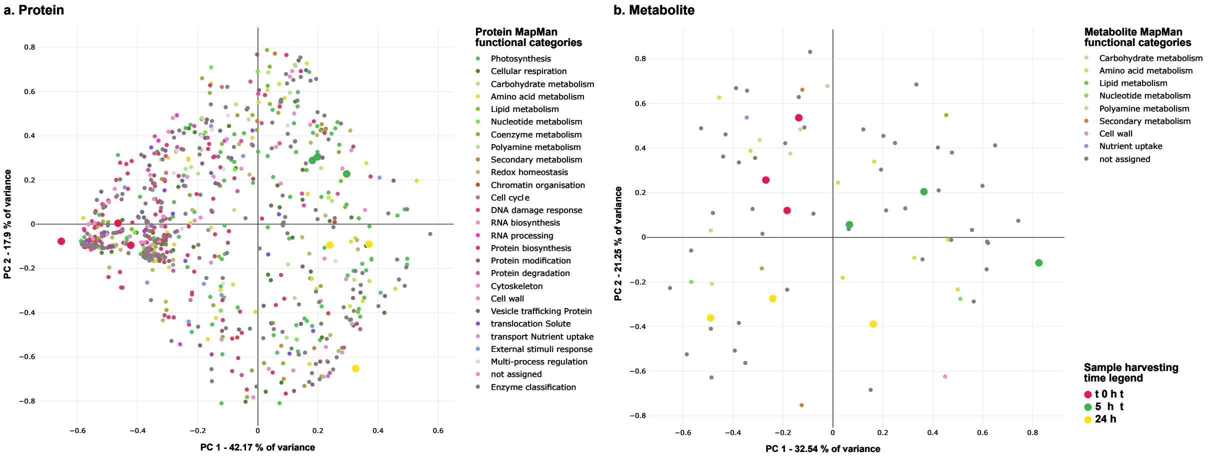

Figure S1. PCA biplots over z-score transformed data from protein (A) and metabolite (B) showing divergence between samples from different harvesting times and the correlation of each individual variables to each displayed component. Protein PCA (A) principal component 1 (PC1) potentially gathers variability related to the photoacclimation to UV-B/C with photosynthesis PHOTOSYSTEM II D2 PROTEIN (psbD) and CHLOROPHYLL A-B BINDING PROTEIN (LHCB4) as some of its highest rank elements. Principal component 2 (PC2) collects early response elements with signaling. WD40 REPEAT PROTEIN (Cre12.g495650.t1.2), carbon metabolism an redox-related NADP MALIC ENZYME 5 (MME5) and TYPE II NADH DEHYDROGENASE (NDA5) and protein damage-associated HEAT SHOCK PROTEIN 70C, E (HSP70C, HSP70E) had the highest loadings within this component. At metabolome level (B), PC1 explain early response with phenolic metabolism protocatechuic and 4-hydroxybenzoic acids; while PC2 joins late accumulated sugars and UV shielding catechin, both related to acclimation. Bigger dots represent individual samples and were colored according to their harvesting time (0, 5, 24 h), while small dots represent individual variables and were colored according to their MapMan category.

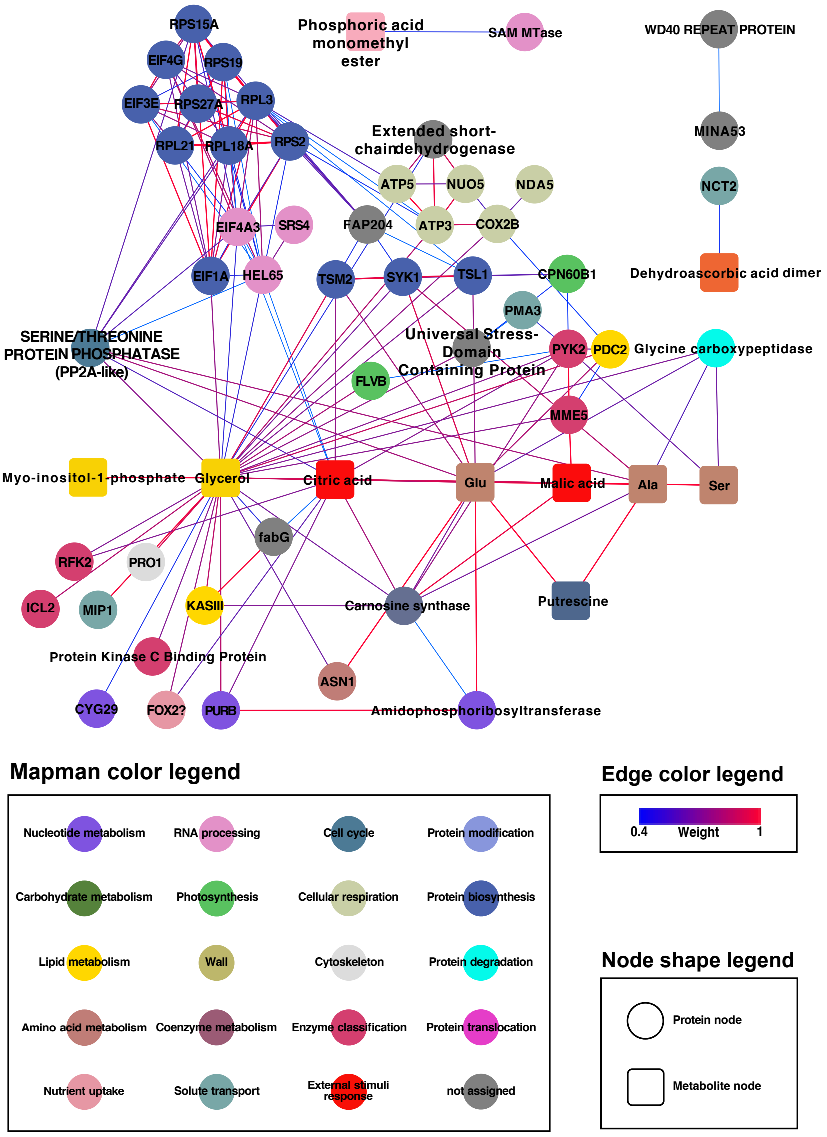

Figure S2. sPLS based STITCH network. STITCH network nodes were colored according to their MapMan categories and shaped according to the omic level element they represented (circle for proteins, quadrangle for metabolites). Edge color was link to interaction confidence (STITCH interaction score). Confidence threshold was set at 0.4 (medium confidence). Network highlighted C3 derived glycerol redox valve central role under UV-C, between protein biosynthesis, respiration modulation, lipid metabolism and oxidative stress response. Same network connected translation related EIF1a, PP2A like (Cre03.g199983.t1.1) and FAP204 to C/N metabolism and translation modulation. Translation/development related MINA53 was also connected into this network to unknow WD40 repeat protein.
